# Supplementary material for: Electrospinning Mo-Doped Carbon Nanofibers as an Anode to Simultaneously Boost Bioelectrocatalysis and Extracellular Electron Transfer in Microbial Fuel Cells
Source: Materials (Basel). 2023 Mar 21;16(6):2479. doi: 10.3390/ma16062479 (PMC10053816; doi:10.3390/ma16062479)
Supplement: Supplementary file 1 [file materials-16-02479-s001.zip › materials-2250956-supplementary.pdf]

# Supplementary Materials

# Electrospinning Mo-doped carbon nanofibers as an anode to simultaneously boost bioelectrocatalysis and extracellular electron transfer in Microbial fuel cells

**Xiaoshuai Wu [*,†], Xiaofen Li [†], Zhuanzhuan Shi, Xiaohai Wang, Zhikai Wang and Chang Ming Li**

Institute of Materials Science and Devices, School of Materials Science and Engineering, Suzhou University of Science and Technology, Suzhou 215011, China;

* Correspondence: wuxiaoshuai365@163.com

† These authors contributed equally to this work.

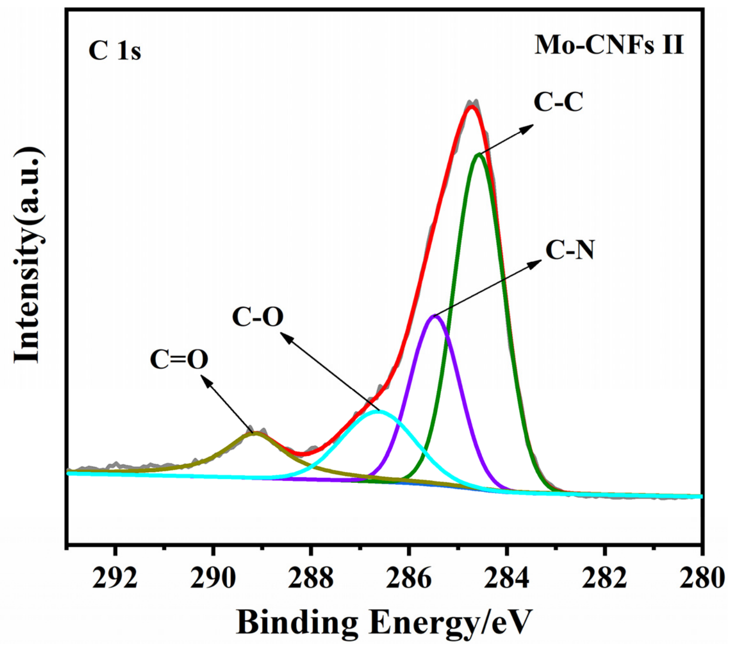


Figure S1. High-resolution C1s XPS spectrum of Mo-CNFs II.

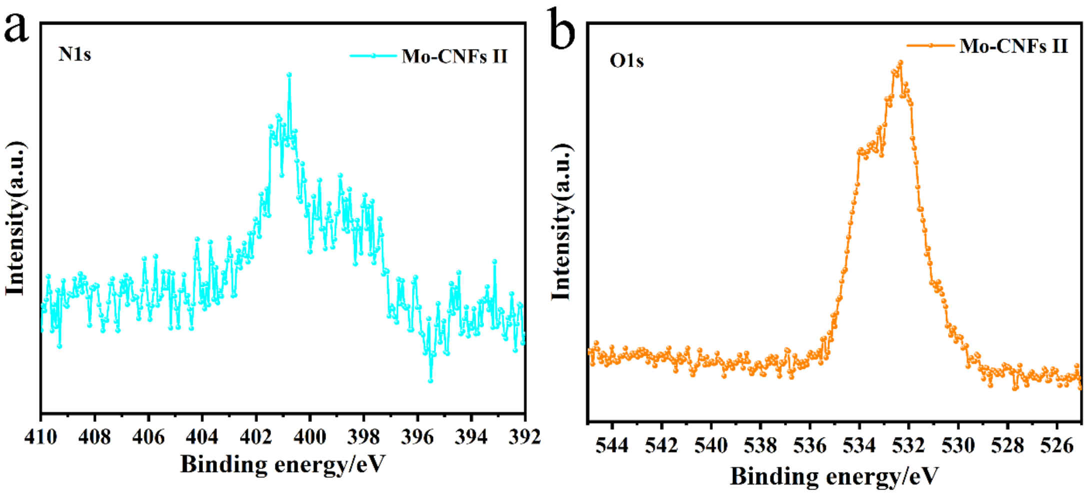


Figure S2. High-resolution N 1s (a) and O 1s (b) XPS spectrum of Mo-CNFs II.

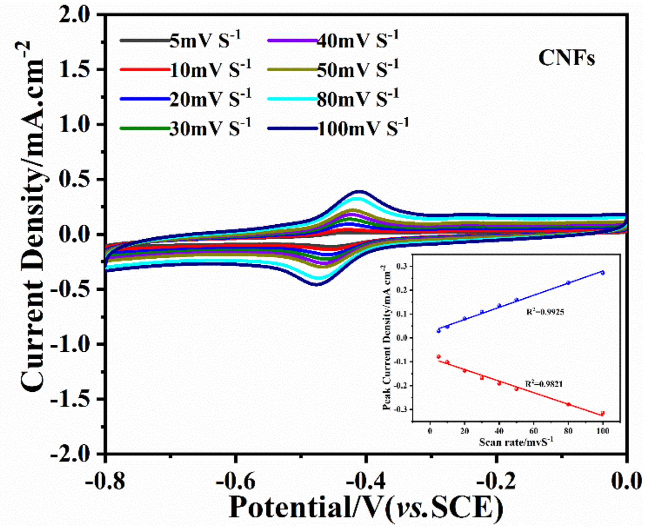


Figure S3. CV curves and Peak current density at different scan rate of the CNFs electrode.

Table S1. A table to compare the performance of the anode materials prepared by this work with previously reported anode materials.

| **Anode materials** | **Reactor configuration** | **Substrate** | **Source of inoculation** | **Performance Pmax ($mW\ m^{-2}$)** | **Reference** |
|---|---|---|---|---|---|
| Mo-CNFs II | Dual chamber | M9 + Lactate | *S.putrefaciens* CN32 | 1287.38 $mW\ m^{-2}$ | This work |
| Fe@CNFs-20 | Dual chamber | M9 + Lactate | *S.putrefaciens* CN32 | 731.05 $mW\ m^{-2}$ | [1] |
| CNTs/CNFs | Dual chamber | artificial wastewater + PBS | mixed bacteria | 362 ± 20 $mW\ m^{-2}$ | [2] |
| GL-$MoS_2$/CC | Dual chamber | PBS + sodium acetate | mixed bacteria | 960.4$mW\ m^{-2}$ | [3] |

| | | | | | |
|---|---|---|---|---|---|
| $Mo_2C$@CF | Dual chamber | Lactate | *S.putrefaciens* CN32 | 1052mW $m^{-2}$ | [4] |
| $Mo_2C$/CCT | Single chamber | sodium acetate | mixed bacteria | 1120 mW $m^{-2}$ | [5] |
| $MnCo_2O_4$@CF | Dual chamber | sodium lactate | mixed bacteria | 945 mW $m^{-2}$ | [6] |
| PANI-T-900 | Dual chamber | Wastewater + acetate | mixed bacteria | 40.4 mW $m^{-2}$ | [7] |
| CNPs/Ti | Single chamber | sodium acetate | mixed bacteria | 616 mW $m^{-2}$ | [8] |
| $Mo_2C$/CNTs | Single chamber | PBBM + glucose | E. coli | 1050 ± 26.4 mW $m^{-2}$ | [9] |
| pristine CC | Dual chamber | M9+sodium lactate | *Shewanella oneidensis* MR-1 | 51.5 mW $m^{-2}$ | [10] |

**References:**


1. Sun, X.; Wu, X. S.; Shi, Z. Z.; Li, X.; Qian, S.; Ma, Y.; Sun, W.; Guo, C.; Li, C. M., Electrospinning iron-doped carbon fiber to simultaneously boost both mediating and direct biocatalysis for high-performance microbial fuel cell. *J. Power Sources* **2022,** 530, 231277.
2. Cai, T.; Huang, M. H.; Huang, Y. X.; Zheng, W., Enhanced performance of microbial fuel cells by electrospinning carbon nanofibers hybrid carbon nanotubes composite anode. *Int. J. Hydrogen Energy* **2019,** 44, (5), 3088-3098.
3. Lou, X.; Liu, Z.; Hou, J.; Zhou, Y.; Chen, W.; Xing, X.; Li, Y.; Liao, Q.; Zhu, X., Modification of the anodes using $MoS_2$ nanoflowers for improving microbial fuel cells performance. *Catal. Today* **2021,** 364, 111-117.
4. Zou, L.; Lu, Z. S.; Huang, Y. H.; Long, Z.-e.; Qiao, Y., Nanoporous $Mo_2C$ functionalized 3D carbon architecture anode for boosting flavins mediated interfacial bioelectrocatalysis in microbial fuel cells. *J. Power Sources* **2017,** 359, 549-555.
5. Zeng, L.; Zhao, S.; Zhang, L.; He, M., A facile synthesis of molybdenum carbide nanoparticles-modified carbonized cotton textile as an anode material for high-performance microbial fuel cells. *RSC Advances* **2018,** 8, (70), 40490-40497.
6. Tahir, K.; Miran, W.; Jang, J.; Maile, N.; Shahzad, A.; Moztahida, M.; Ghani, A. A.; Kim, B.; Lee, D. S., $MnCo_2O_4$ coated carbon felt anode for enhanced microbial fuel cell performance. *Chemosphere* **2021,** 265, 129098.
7. Lascu, I.; Locovei, C.; Bradu, C.; Gheorghiu, C.; Tanase, A. M.; Dumitru, A., Polyaniline-Derived Nitrogen-Containing Carbon Nanostructures with Different Morphologies as Anode Modifier in Microbial Fuel Cells. *Int. J. Mol. Sci.* **2022,** 23, (19), 11230.
8. Pu, K.-B.; Zhang, K.; Guo, K.; Min, B.; Chen, Q.-Y.; Wang, Y.-H., Firmly coating carbon nanoparticles onto titanium as high performance anodes in microbial fuel cells. *Electrochim. Acta* **2021,** 399, 139416.
9. Wang, Y.; Li, B.; Cui, D.; Xiang, X.; Li, W., Nano-molybdenum carbide/carbon nanotubes composite as bifunctional anode catalyst for high-performance Escherichia coli-based microbial fuel cell. *Biosens Bioelectron* **2014,** 51, 349-355.
10. Liu, X.; Zhao, X.; Yu, Y.-Y.; Wang, Y.-Z.; Shi, Y.-T.; Cheng, Q.-W.; Fang, Z.; Yong, Y.-C., Facile

fabrication of conductive polyaniline nanoflower modified electrode and its application for microbial energy harvesting. *Electrochim. Acta* **2017,** 255, 41-47.
